# Ecobiology of *Haemagogus leucocelaenus* arbovirus vector in the golden lion tamarin translocation area of Rio de Janeiro, Brazil

**DOI:** 10.1038/s41598-023-39629-x

**Published:** 2023-08-12

**Authors:** Sergio Lisboa Machado, Cecilia Ferreira de Mello, Shayenne Olsson Freitas Silva, Jeronimo Alencar

**Affiliations:** 1 Laboratory of Molecular Diagnosis and Hematology, Universidade Federal do Rio de Janeiro, Rio de Janeiro, 21941-901 Brazil; 2 Graduate Program in Animal Biology, Instituto de Biologia, Universidade Federal Rural do Rio de Janeiro, Seropédica, 23890-000 Brazil; 3 Diptera Laboratory, Instituto Oswaldo Cruz (FIOCRUZ), Rio de Janeiro, 21040-360 Brazil

**Keywords:** Ecology, Zoology

## Abstract

Significant pathogens that have resurfaced in humans originate from transmission from animal to human populations. In the Americas, yellow fever cases in humans are usually associated with spillover from non-human primates via mosquitoes. The present study characterized the prevalence of the yellow fever vector *Haemagogus leucocelaenus* in Rio de Janeiro, Brazil. The Atlantic Forest fragment chosen is an area of translocation of the golden lion tamarin (*Leontopithecus rosalia*), where 10 ovitraps were installed to collect mosquito eggs in Fazenda Três Irmãos, at Silva Jardim city, from March 2020 to October 2022. A total of 1514 eggs were collected, of which 1153 were viable; 50% belonged to medically important mosquito species and 24% to the yellow fever vector species, *Hg. leucocelaenus*. The months of December 2020 (n = 252), November 2021 (n = 188), and January 2022 (n = 252) had the highest densities of this vector. *Haemagogus leucocelaenus* was positively correlated with temperature (r = 0.303) and humidity (r = 0.48), with eggs hatching up to the 15th immersion with higher abundance of females. Implementing mosquito monitoring for arbovirus activity can help protect both the golden lion tamarin and human populations from the threat of arbovirus transmission.

## Introduction

Several diseases that have resurfaced or emerged in humans, such as ebola, sudden acute respiratory syndrome (SARS), influenza, primate malaria, yellow fever, and leptospirosis, originate from the transmission of pathogens from animal to human populations^1,2^. Yellow fever virus (YFV) is an acute febrile infectious disease transmitted by vector mosquitoes, with two transmission cycles: wild (when transmission occurs in rural or forest areas) and urban (when transmission occurs in cities)^3,4^. Even with a safe vaccine, YFV continues to cause morbidity and mortality in thousands of people in South America and Africa^4^. The recent introduction of YFV in the Americas makes neotropical non-human primates (NHP) highly susceptible to infection^5^.

The golden lion tamarin (*Leontopithecus rosalia*; GLT) is a critically endangered primate found only in the Brazilian Atlantic Forest, mainly in the São João River basin, which is a fragment with limited forest connection to other fragments^6^. Golden lion tamarins are small arboreal primates that are particularly vulnerable to arbovirus infections due to their low genetic diversity and lack of prior exposure to many infectious agents. In addition, the species is susceptible to habitat loss and fragmentation, which can increase the risk of disease transmission^7–9^. Deforestation due to anthropic activities has reduced the population to a few hundred individuals in isolated forest fragments. Conservation work has resulted in the recovery of some GLT populations; however, YFV outbreaks threaten the conservation and maintenance of this species in nature^10^.

The Atlantic Forest fragment chosen for the sampling is an area of translocation of the GLT. Projects to reintroduce the species and establish protection areas, such as in Poço das Antas, Silva Jardim, resulted in an increase in the population of this species. However, significant reductions have recently been observed: population numbers declined from 3700 individuals in 2014 to 2516 in 2018, which is equivalent to a 32% decrease. In May 2018, there was a report of the first death of a wild GLT due to YFV in decades. In response to this event, a new count carried out in the Poço das Antas reserve determined that a significant loss of these tamarins occurred inside the larger forest fragment rather than on its edges^10^. In forest areas of the Americas, YFV is transmitted by infected mosquitos belonging to the genus *Haemagogus* Williston, 1896 and *Sabethes* Robineau-Desvoidy, 1827^11^. *Haemagogus* mosquito species, whose larval habitats are stumps of trees, bromeliads, and bamboo, are diurnal and are frequently found near the treetops. This makes GLTs a blood-meal source option, putting them at risk for YF infection^12,13^. Regarding blood source preference, *Hg. leucocelaenus* Dyar & Shannon, 1924 prefer NHP, although this species and *Hg. janthinomys* Dyar, 1921*/capricornii* Lutz, 1904 are eclectic in their host preference for biting other animals, such as cattle, birds, dogs, rodents, and horses, adapting to modified environments. *Haemagogus janthinomys/capricornii* usually habitually stay in the canopy of trees and bite around noon, while *Hg. leucocelaenus* usually circulates on the forest floor and has a predominant afternoon biting activity^14–16^.

The population at risk of contracting the disease and becoming ill includes those who are not vaccinated and are subject to bites from infected vector mosquitos in forest areas within the endemic area of YFV^17^. Previously, the YF vaccine was offered only to some regions of the country with disease cases. With the expansion of the virus and an increase in the number of areas reporting cases, the Ministry of Health has been gradually expanding the recommended vaccination areas. Although the YF vaccine has been expanded to all states in the Northeast, vaccination is now recommended throughout the country^18^.

From November 2016 to 2018, Brazil experienced a significant YF outbreak, considered one of the largest the country had, with 2,058 confirmed cases and 689 human deaths. This outbreak was concentrated mainly in rural areas, particularly in Brazil’s southeast and southern regions, where the virus had not been previously detected^16^. YFV expansion started in the Amazon region, spreading from the Central-West region, home to a savanna-like biome, to Atlantic Forest fragments of Minas Gerais (MG), Espírito Santo (ES), Rio de Janeiro (RJ), and São Paulo (SP) states^17^. Cases have also been reported in urban areas, including on the outskirts of major cities like São Paulo and Rio de Janeiro^16,19^.

The dispersion of arboviruses over time and space is primarily influenced by their vectors' behavior. *Haemagogus janthinomys/capricornii* and *Hg. leucocelaenus* are considered primary YFV vectors due to their wide distribution, high abundance, and natural infection rates^20^. Another element influencing the spread of diseases carried by vectors is the climate. A change in climatic factors can directly impact the geographical distribution and incidence of vector-borne diseases^21–24^. Hence, the current study aims to characterize the prevalence and biodiversity of *Haemagogus* spp., focusing on *Hg. leucocelaenus*.

## Results

### Seasonal abundance of Culicidae eggs

**Figure 1:**
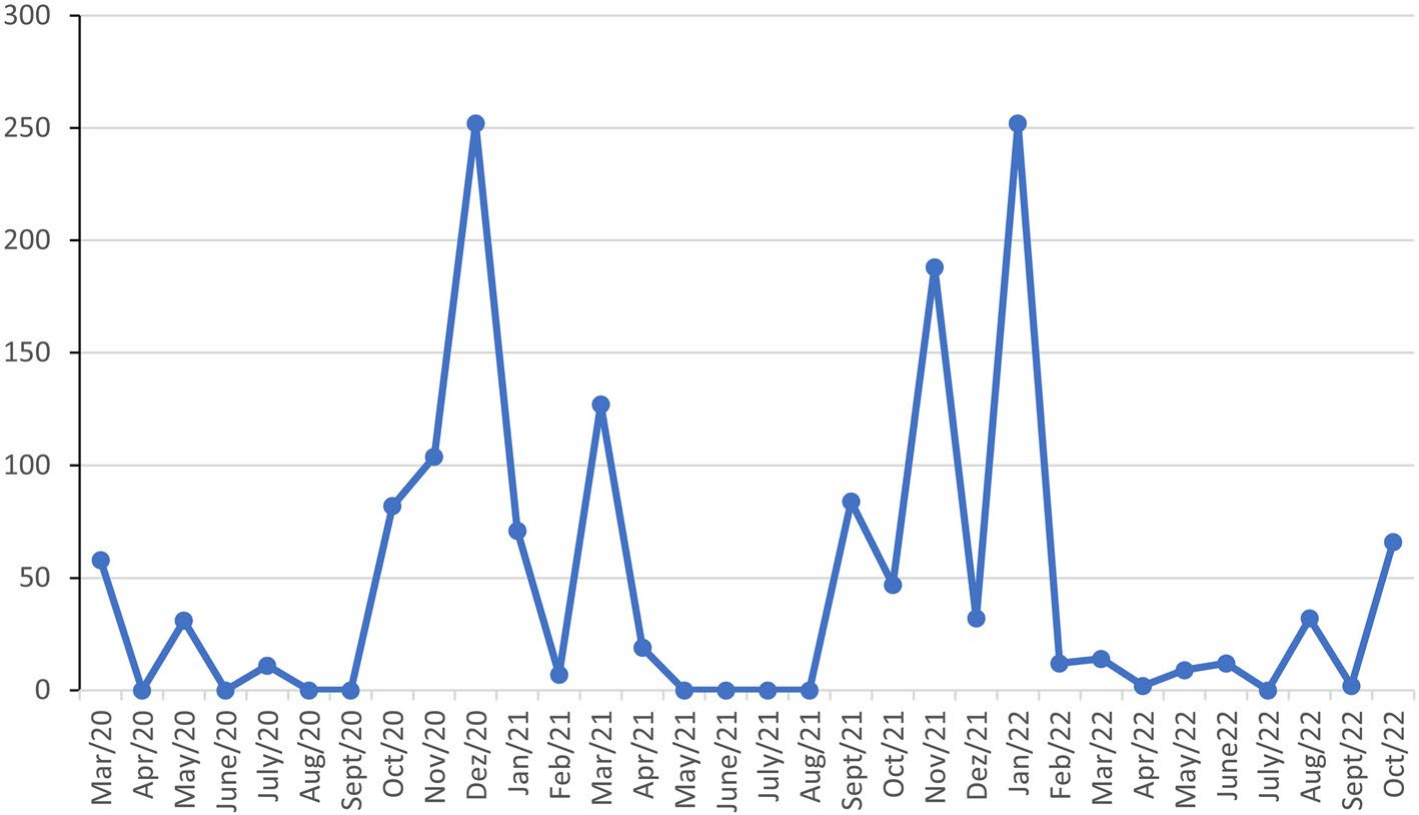
Fluctuation in the number of mosquito eggs collected per month, from March 2020 to October 2022, at Fazenda Três Irmãos, Silva Jardim, Rio de Janeiro, Brazil.

During the collection period from March 2020 to October 2022, the number of eggs showed significant peaks in December 2020 (n = 252), November 2021 (n = 188), and January 2022 (n = 252)—all comprising summer months of the rainy season. December 2020 had the highest average temperature for the year (22.4 °C) as well as a high level of rainfall (4.3 mm) compared to the other months of 2020. January 2022 was also marked by the highest average temperature for the year (22.8 °C) (INPE 2023). The lowest abundances were observed in May and August 2021, with the number of eggs equal to zero—the autumn and winter months of the dry season—and these months had low average temperatures and rainfall equal to zero (Fig. 1).

**Figure 2:**
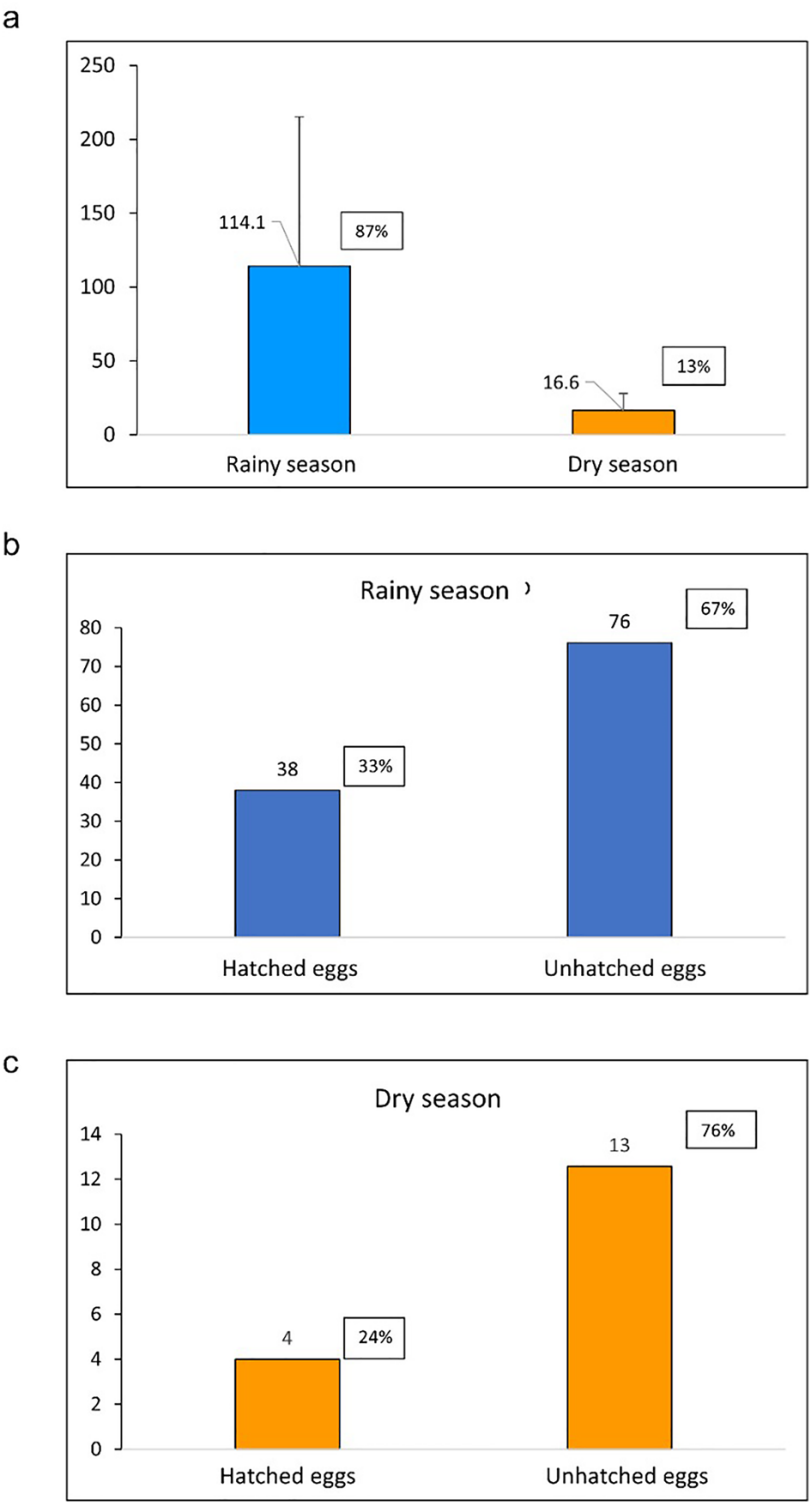
Number of mosquito eggs collected in the dry and rainy seasons (**A**). Number of hatched and unhatched eggs in the rainy (**B**) and dry season (**C**).

There was a significant difference (p ≤ 0.01) between the total number of eggs collected in the dry and rainy seasons (p = 0.0026), with more eggs collected in the rainy season (87%). The dry period showed the greatest difference between the number of hatched and unhatched eggs (52%), when compared to the rainy season (33%). In both seasons the number of unhatched eggs was higher. More hatched eggs were found in the rainy season. Of the total eggs collected, the percentage of hatched eggs in the rainy season was 29%, while in the dry season, it was only 3%. In this case, seasonality seems to have influenced egg hatching. Rainfall likely stimulated the hatching of a larger number of eggs in a shorter time (Fig. 2).

**Figure 3:**
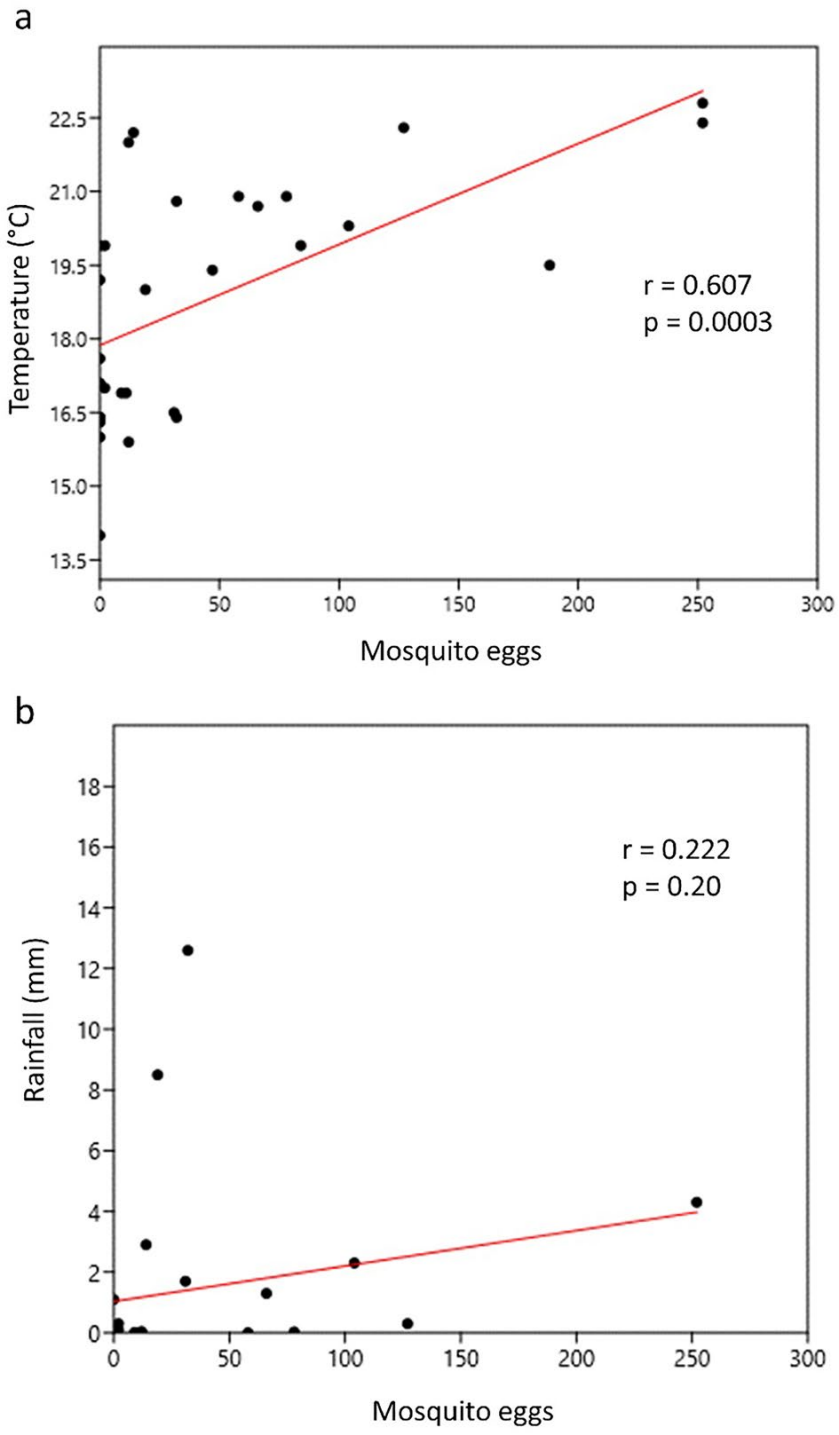
Linear regression between the number of eggs collected and the environmental variables temperature (**A**) and rainfall (**B**).

There was a positive and significant correlation (r = 0.607) between the number of Culicidae eggs and temperature and a positive but weak correlation between the number of eggs and rainfall (r = 0.222) (Fig. 3). There was a positive correlation between *Hg. leucocelaenus* and the environmental variables temperature (r = 0.303) and humidity (r = 0.48).

### Influence of multiple immersions

The highest percentage of *Hg. leucocelaenus* eggs hatched was observed in the 1st immersion (49%), and the second-highest was in the 3rd immersion (17%). Egg hatching of this species was observed until the 15th immersion. Eggs from females hatched until the 13th immersion, while males hatched until the 15th; therefore, the last eggs hatched were from males. In the initial immersions, more males than females were hatched, as follows: 1st immersion (M = 50%; F = 48%), 2nd immersion (M = 63%; F = 62%), 3rd immersion (M = 82%; F = 78%). This probably happened because males need more time after immersion to complete their development since, on the first day after emergence, they undergo a 180-degree rotation of the entire abdominal extremity from segment VIII, including the genitalia^25,26^.

With regard to *Hg. leucocelaenus,* the species with the highest abundance, the number of females and males was very similar, with 157 females (56%) and 125 males (44%), resulting in a difference of 11% more females. The months with the highest number of females were March, November and December 2020, and March and September 2021. The only month with a higher number of males was August 2022. May and October 2020 had an equal number of males and females.

### Species that performed oviposition in the same ovitrap

Eggs from *Hg. leucocelaenus* and *Hg. janthinomys/capricornii* were found together in three of the 10 ovitraps (5, 6, and 8) in Fazenda Três Irmãos. Most eggs belonged to *Hg. leucocelaenus,* except for trap number 5, which had more *Hg. janthinomys/capricornii* eggs. Eggs of the species *Hg. leucocelaenus* and *Ae. terrens* were also observed in the same ovitrap (3).

## Discussion

According to a study that combined vector suitability, the presence of NHP host reservoirs, and population density, Rio de Janeiro is considered one of the risk areas for mapping YFV transmission and spillover in the Southeastern Atlantic Forest Biome^27^. The presence of wild mosquito vectors in forest areas of Rio de Janeiro was previously observed in other studies^12,28^. In the present study, the vector with the highest abundance was *Hg. leucocelaenus*. However, *Hg. janthinomys/capricornii* and *Ae. terrens* Walker, 1956, were also present. The occurrence of these species raises concern because *Hg. leucocelaenus* and *Hg. janthinomys/capricornii* have been reported as positive for YFV in Rio de Janeiro^19,29^.

The rainy season had the highest densities of vector populations, mainly in December 2020, November 2021, and January 2022; these results corroborate those of other studies, which reported high peaks during the rainy season^28,30^. A positive and significant correlation between the number of Culicidae eggs and temperature was also observed in Casimiro de Abreu, a city located 36.8 km from our study area^12^. Similarly, there was a positive correlation between the vector species *Hg. leucocelaenus* and *Ae. terrens* with the environmental variables of temperature and humidity. The species *Haemagogus janthinomys/capricornii* had a positive and statistically significant correlation with rainfall. The dynamics of arbovirus transmission appear to be significantly influenced by environmental conditions. High temperatures have been observed to boost mosquito population sizes, and mosquito density has been strongly linked to the spread of the diseases they carry, such as YF. Climate factors like rainfall appear to precede zika and Chikungunya epidemics^31,32^.

The different survival mechanisms mosquitoes have developed throughout their life histories account for their evolutionary success in tropical and temperate climates^33^. Some species exhibit the strategy of egg dormancy (either diapause or quiescence) as a reproductive tactic, which enables them to endure long periods in environments unsuitable for hatching^34^. *Haemagogus leucocelaenus* hatched until the 15th immersion, and the need for multiple immersions for egg hatching in this species was also observed by Silva et al. 2018, who showed that *Hg. leucocelaenus* demonstrated installment hatching up to the 37th immersion^35^.

The selection of ovipositing sites by females is the primary factor responsible for the distribution of mosquitoes^36^. Eggs from epidemiologically important species were found in the same breeding site (ovitrap); they belonged to *Hg. leucocelaenus*, *Hg. janthinomys/capricornii* and *Ae. terrens*. The species that were observed sharing the same ovitrap most frequently were *Hg. leucocelaenus* and *Hg. janthinomys/capricornii*, probably related to the fact that they are co-generic. *Haemagogus leucocelaenus* and *Ae. terrens* were also observed sharing the same breeding site; however, this scenario was less frequent. These observations corroborate Silva et al.^12^, who observed that these species shared the same breeding site and showed a positive correlation. The co-occurrence of these vector species may imply an overlapping of the etiological agent they transmit, such as YFV (*Hg. leucocelaenus*) and Chikungunya virus (*Ae. terrens*)^37,38^.

Understanding the presence, seasonal abundance of sylvatic vectors, and possible contact with reservoirs and humans in fragments of the Atlantic Forest in Rio de Janeiro is of great interest to public health since it can help predict possible areas of YF spillover. Knowledge regarding mosquito vector populations plays a critical role in providing a framework to protect wild primates and humans’ health along with the preservation of the ecosystems we share.

## Conclusions

Monitoring arbovirus activity in mosquitoes and primates is essential for detecting and responding to potential outbreaks. Therefore, it is also important to consider the broader ecological context of disease transmission, including the role of wildlife and human activities in shaping the risk of disease emergence. Deforestation and changes in land use can increase the frequency of contact between wildlife, mosquitoes, and humans, increasing the risk of disease transmission. Understanding the ecobiology of *Hg. leucocelaenus* and its role as an arbovirus vector in the GLT translocation area is essential for effective conservation and disease management efforts. Implementing mosquito monitoring for arbovirus activity can help protect both the GLT and human populations from the threat of arbovirus transmission.

## Materials and methods

### Ethics statement

The study was carried out in accordance with scientific license number 44333 provided by the Ministério do Meio Ambiente (MMA), Instituto Chico Mendes de Conservação da Biodiversidade (ICMBio), Biodiversity Information and Authorization System (SISBIO) in Atlantic Forest areas of Rio de Janeiro with the agreement of the properties or the state government where the mosquitoes were captured. All members of the collection team were vaccinated against YFV and aware of the potential risks in the study areas, no animals or humans were used in the development of this study.

### Study area

**Figure 4:**
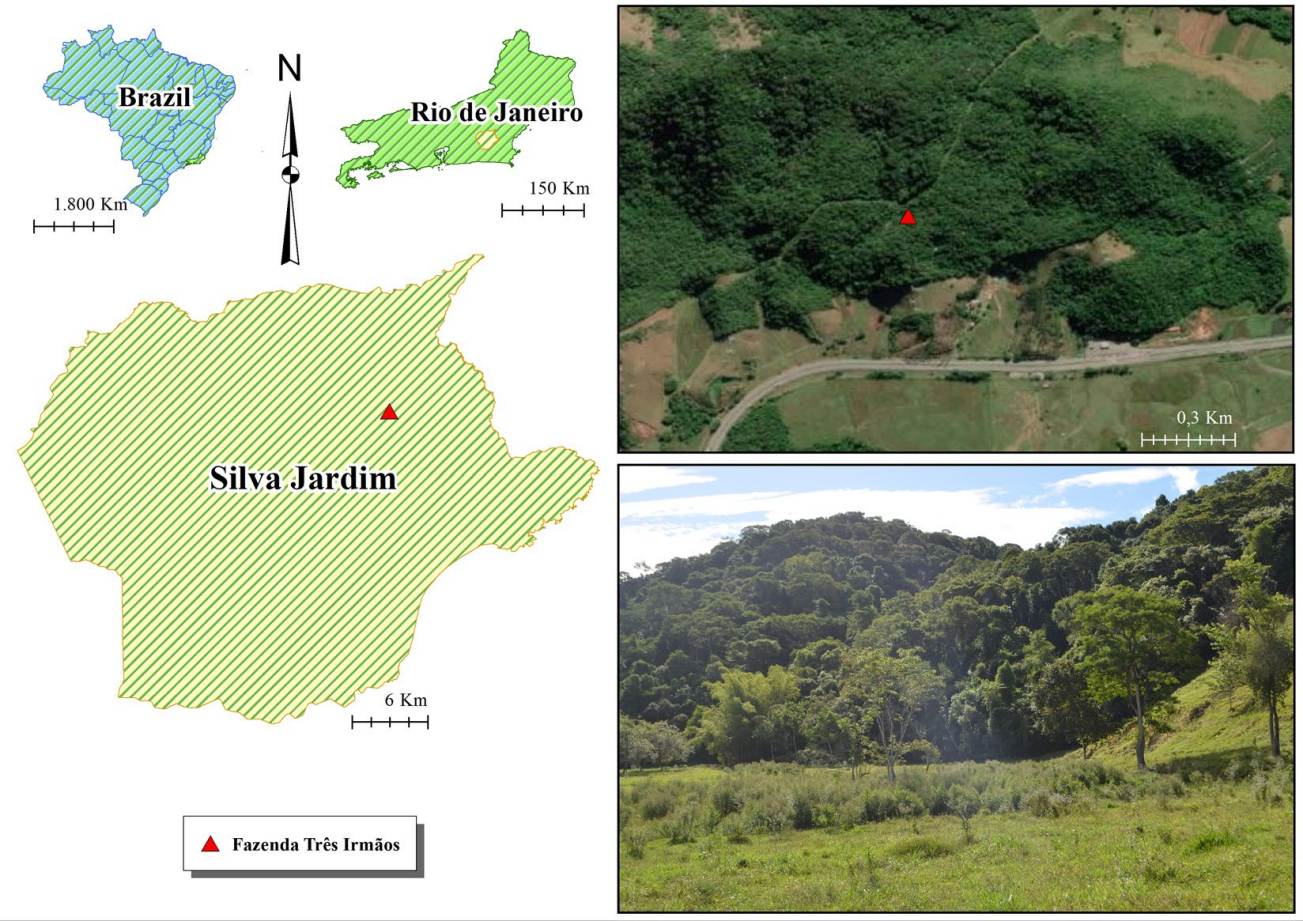
Map of the study site, municipality of Silva Jardim, Rio de Janeiro, Brazil.

The study was conducted in Fazenda Três Irmãos (22°30′46.9" S; 42°20′13.0" W), Silva Jardim, Rio de Janeiro, Brazil (Fig. 4). Rio de Janeiro is located in the middle of the Atlantic Forest Biome, representing one of the areas with the greatest diversity of this biome in the country, with unique mountain ranges and coastal plains^39^. The geomorphological units that make up this state provide a diversity of vegetational landscapes and, consequently, an expressive variety of habitats and species richness, including several endemic ones.

### Collection and raising of immature Culicidae

The collection period was between March 2020 and October 2022. One trap was placed at each collection site, and a total of 10 ovitraps were distributed. Each trap had three wooden oviposition paddles that measured 2.5 cm by 14 cm and had textured surfaces to help with oviposition. The paddles were clipped vertically into the traps. In an effort to mimic the microecosystem seen at natural breeding locations, traps were loaded with 300 ml of fresh water from nearby bodies of water and about 100 g of leaf litter. The ovitraps were affixed to the trees at a height of 2.5 m using nylon ropes and wire. Every 20 days, the traps were checked, and after each inspection, the wooden paddles carrying the eggs were removed and brought to the lab for quantification before being replaced with fresh paddles for subsequent collections. Paddles were collected from the field ovitraps and brought to the Oswaldo Cruz Institute's Diptera Laboratory, where they underwent egg counting and were submerged in transparent trays filled with dechlorinated water. The trays with viable eggs were placed in a controlled experimental environment in a laboratory greenhouse with a thermoperiod controlled at a temperature of 28 °C ± 1 °C, relative air humidity of 75–90%, and a 12-h day/12-h night cycle. After three days, the paddles were taken out of the water and allowed to air dry for an additional three days to count the newly hatched larvae. The larvae were given TetraMint fish food (Tetra, Blacksburg, VA) and monitored daily. These conditions allowed us to keep the specimens alive until adulthood for species determination, according to the methodology described by Alencar et al.^40^.

Adult mosquitoes from the eggs collected in the field were identified at the species level through direct observation of their morphological characters using a stereo microscope (Zeiss) and dichotomous keys elaborated by Arnell (1973) and Forattini (2002)^11,41^. After the specific determination, all specimens were incorporated into the Entomological Collection of the Oswaldo Cruz Institute, Fiocruz.

### Statistical analysis

The data were tested for normality, and the t-test was used to compare the number of Culicidae eggs collected in the rainy and dry seasons. Correlations between the number of eggs and vector species with climatic and environmental variables were assessed from 2020 to 2022. The meteorological data, including average maximum, minimum, and annual temperatures (°C), average relative humidity (%), and precipitation (mm), were retrieved from the Centro de Previsão de Tempo e Estudos Climáticos [Center for Weather Forecasting and Climate Studies]—CPTEC/INPE (INPE 2023).

### Supplementary Information

Supplementary Information.

## Data Availability

The data for this study is stored at Fundação Oswaldo Cruz and can be made available by the corresponding author (JA) upon request.
